# Effect of Ornamental Stone Waste Incorporation on the Rheology, Hydration, Microstructure, and CO_2_ Emissions of Ordinary Portland Cement

**DOI:** 10.3390/ma15020401

**Published:** 2022-01-06

**Authors:** Taylana Piccinini Scolaro, Laura Silvestro, Artur Spat Ruviaro, Afonso R. G. de Azevedo, Sergio Neves Monteiro, Fernando Pelisser

**Affiliations:** 1 Department of Civil Engineering, Federal University of Santa Catarina (UFSC), Rua João Pio Duarte Silva 151, Florianópolis 88040-900, Brazil; taylanaps@hotmail.com; 2 Laboratory of Nanotechnology Applications in Civil Construction (LabNANOTEC), Department of Civil Engineering, Federal University of Santa Catarina (UFSC), Rua João Pio Duarte Silva 151, Florianópolis 88040-900, Brazil; laura.silvestro@posgrad.ufsc.br (L.S.); artur.spat@posgrad.ufsc.br (A.S.R.); pelisser@hotmail.com (F.P.); 3 Civil Engineering Laboratory (LECIV), State University of Norte Fluminense Darcy Ribeiro (UENF), Av. Alberto Lamego 2000, Rio de Janeiro 28013-602, Brazil; 4 Department of Materials Science, Military Institute of Engineering (IME), Square General Tibúrcio 80, Rio de Janeiro 22290-270, Brazil; snevesmonteiro@gmail.com

**Keywords:** sustainable cementitious composites, ordinary Portland cement, ornamental sandstone waste, rheology, hydration, strength, CO_2_ emission

## Abstract

The ornamental stone industry generates large amounts of waste thus creating environmental and human health hazards. Thus, pastes with 0–30 wt.% ornamental stone waste (OSW) incorporated into ordinary Portland cement (OPC) were produced and their rheological properties, hydration kinetics, and mechanical properties were evaluated. The CO_2_ equivalent emissions related to the pastes production were estimated for each composition. The results showed that the paste with 10 wt.% of OSW exhibited similar yield stress compared to the plain OPC paste, while pastes with 20 and 30 wt.% displayed reduced yield stresses up to 15%. OSW slightly enhanced the hydration kinetics compared to plain OPC, increasing the main heat flow peak and 90-h cumulative heat values. The incorporation of OSW reduced the 1-, 3-, and 28-days compressive strength of the pastes. Water absorption results agreed with the 28 days compressive strength results, indicating that OSW increased the volume of permeable voids. Finally, OSW incorporation progressively reduced the CO_2_ emission per m^3^ of OPC paste, reaching a 31% reduction for the highest 30 wt.% OSW content. Overall, incorporating up to 10 wt.% with OSW led to pastes with comparable fresh and hardened properties as comported to plain OPC paste.

## 1. Introduction

Sandstone composed mainly of quartz is commonly extracted as an ornamental plate. Its use as a building material generates significant ornamental sandstone waste (OSW) in the quarries [1,2]. It is estimated that 25% of the extracted ornamental sandstone become waste due to cutting and polishing [3]. These OSW powders are waste that can cause environmental and human health hazards. Indeed, fine industrial wastes can be carried by the wind and the water. They contaminate land and waterways resulting in human breathing difficulties [4,5]. Utilizing local wastes, such as OSW, as a partial replacement to conventional cementitious materials can minimize environmental and human health hazards, decrease CO_2_ emissions, and even reduce material costs [5]. Furthermore, according to Ramav [6], sandstone corresponds to about 20% of the continental rocks, emphasizing the potential of using sandstone waste to produce cementitious materials. In 2016 the world production of ornamental stones reached a value of 145 Mt/year. In this scenario, Brazil stood out as the fourth-largest producer of this sector [7]. Thus, these data show that ornamental stone waste is also a national environmental problem.

Various studies have used stone waste (e.g., marble and granite) as an alternative binder or aggregate for making green construction materials [8,9,10,11]. Some researchers reported the use of sandstone waste in cementitious materials. For example, Kumar et al. [3] used quartz sandstone to replace coarse aggregate in cement concretes. The concretes with sandstone presented, although presenting lower strength, were equivalent to the control concrete, with considerable carbonation resistance and decreased porosity up to 40% substitution. Arif et al. [1] reported that sandstone slurry from Rajasthan, India, can replace up to 15% of the total aggregate, thus reaching compressive and flexural strength values comparable to the control concrete. Mundra et al. [12] produced concretes with partial replacement of river sand by sandstone cutting waste from India. The authors observed that the sandstone might be incorporated up to 10%, with water/cement (w/c) ratio of 0.35, and up to 30% replacement content, with w/c of 0.40 and 0.45, without compressive strength losses. This improvement is due to de filler effect of the sandstone cutting, which contributes to the formation of a dense matrix. Basu et al. [5] partially replaced Portland Pozzolana cement with sandstone slurry in self-compacting concrete (SCC). Although the incorporation of sandstone reduced the strength and increased water absorption, it was possible to substitute sandstone slurry up to 15% in SCC. In another study, Basu et al. [13] reported an increase in the depth of carbonation and chloride penetration, supported by increased porosity, which was verified using mercury intrusion porosimetry in SCC with sandstone from Rajasthan, India. Moreover, the durability test results were comparable to the control concrete [13].

The aforementioned studies indicate the feasibility of OSW as a partial substitution for aggregate or cement in cementitious materials. However, the physical–chemical properties of OSW may vary according to their composition, affecting the properties of pastes, mortars, or concretes produced with them [3,12]. Thus, it is fundamental to investigate sandstone from different regions of the globe. Furthermore, the rheology and the hydration of OPC composites with cement replacement with sandstone has been seldom studied. This study also addresses CO_2_ emissions, contributing to sustainable cementitious composites. In this sense, the current study investigates the incorporation of a specific OSW from southern Brazil in cementitious materials.

It is well-known that the cement industry has significant environmental impacts since its production involves high energy consumption and carbon dioxide (CO_2_) emissions. In fact, approximately 0.7 tons of CO_2_ is released to produce 1 ton of ordinary Portland cement (OPC) clinker [14]. As a result, OPC production is responsible for about 13% of the world’s industrial energy and about 8% of the CO_2_ global emissions [15,16].

The present work addresses the following points (i) reduction of environmental hazards by using waste material in the construction sector and (ii) production of sustainable cement pastes by reducing the OPC consumption and, consequently, the CO_2_ emissions. Thus, it aims to investigate the effect of replacing OPC by 10, 20, and 30 wt.% OSW on the paste’s rheological properties, hydration kinetics, and mechanical behavior. Besides, it will evaluate the CO_2_ equivalent emissions related to pastes production.

## 2. Materials and Methods

### 2.1. Materials

**Figure 1:**
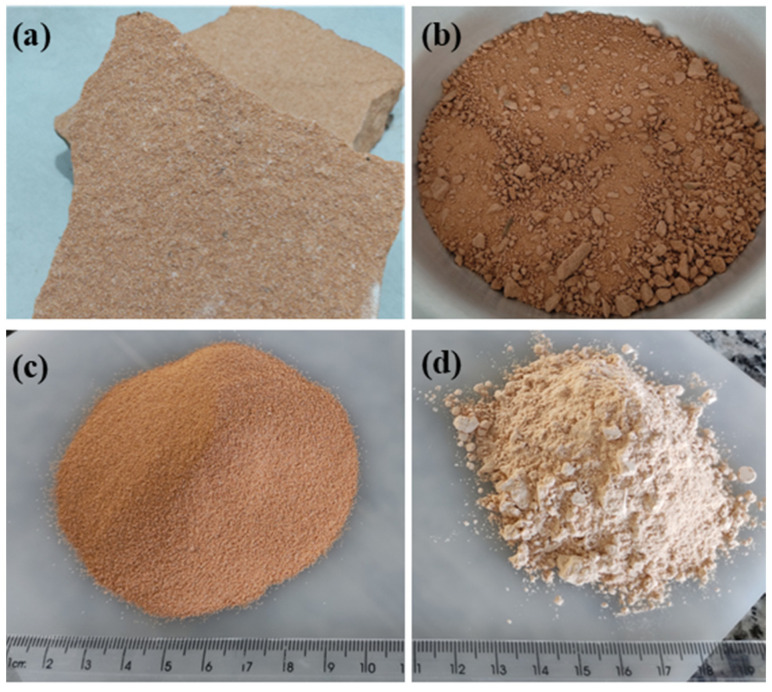
Ornamental sandstone waste (OSW) powder preparation (**a**); manually comminuted (**b**); particles < 30 µm (**c**); and OSW as filler (**d**).

The basic materials used in this work were an OPC available in Brazil as CPV-ARI [17], equivalent to Type III by ASTM C150 [18], and a natural OSW from a quarry in Sapucaia do Sul, RS, Brazil. The OSW was collected in the form of patches. Initially, the waste was manually comminuted and ground for 3 h at 300 rpm in a planetary ball mill (Pulverisette 6, Fritsch, Idar-Oberstein, Germany), as illustrated in Figure 1.

Note that this griding time was adopted because it resulted in a material with a similar average particle size to the cement used. It is important to consider that, although longer grinding times might result in a finer waste, they would require greater energy consumption for its processing, which would go against the development of more sustainable cement-based materials that are the aim of this study. Furthermore, a polycarboxylate-based superplasticizer (MC-PawerFlow 4000, Mc-Bauchemie, Philippines) was used in the paste preparation, with a density of 1.12 g/cm^3^, a solids content of 42.1%, and pH 6.15.

### 2.2. Characterization of Basic Materials

The composition of both OPC and OSW was obtained by X-ray fluorescence (XRF) in an EDX-700 Shimadzu, Osaka, Japan. The OSW particles were analyzed by scanning electron microscopy (SEM) in a model JSM-6390 LV Joel microscope, JOEL, Osaka, Japan. For the analysis, OSW powder sample was placed on a carbon tape and covered with a thin gold layer.

### 2.3. X-ray Diffraction (XRD)

XRD analysis was conducted in a model Miniflex II, Rigaku, Osaka, Japan, equipment operating at 30 kV/15 mA using CuKα radiation, with 2θ angle from 3 to 90° and 0.05° of step size. The phase identification was carried out using the International Centre for Diffraction Data (ICDD) database.

### 2.4. Mix Proportions

Initially, the paste with only OPC was defined (designated as REF), with a water to binder ratio (w/b ratio) of 0.5 by weight and 0.025% of superplasticizer content by weight of binder, to ensure proper flowability (mini-slump of 81 mm). Then, the OPC was incorporated with OSW in levels of 10, 20, and 30 wt.%. The superplasticizer (SP) content of pastes with OSW was adjusted to reach an equivalent flowability (i.e., a mini-slump of 80 ± 5 mm). A similar approach was used in previous studies that evaluated the incorporation of waste as a partial replacement of OPC [19,20,21]. Table 1 shows the detailed pastes’ compositions.

### 2.5. Sample Preparation

The pastes’ mixing procedure consisted of the following steps: (i) powdery material was previously homogenized by hand for 2 min; (ii) dry materials were placed in the mixer; (iii) water and the superplasticizer were added; and (iv) mechanical mixing was conducted at 1600 rpm for 2 min.

### 2.6. Testing Methods

#### 2.6.1. Calorimetry Test

The influence of the incorporated materials on the hydration kinetics of OPC was evaluated by isothermal calorimetry (TAM Air calorimeter, TA Instruments, New Castle, USA). After the sample preparation, about 10 g of paste was placed in the calorimeter container. The temperature was kept at 23 °C, and the measurements were recorded for 90 h.

#### 2.6.2. Slump and Rheometer Testes

The mini-slump test was performed according to Kanto [22]. Rotational rheometry test was conducted using a Haake MARS III (Thermo Fisher Scientific, Bellefonte, PA, USA) rheometer with a vane geometry (16 mm in diameter × 22 mm in height) with four blades. Both tests were performed in samples with 25 mL at 23 °C, applying the procedure described by Matos et al. [23]. The dynamic yield stress (*τ*_0_) and equivalent plastic viscosity (µ_eq_) were calculated considering the decreasing part of the flow curve using Equations (1) and (2), corresponding to the Herschel–Bulkley model and the equation proposed by Larrard et al. [24], respectively.

(1) $$\tau =\tau_{0}+K\cdot {\dot{ɣ}}^{n}$$

(2) $${µ}_{\mathrm{eq}}=\frac{3K}{n+2}\times {\left({\dot{ɣ}}_{max}\right)}^{n-1}$$

where τ is the shear stress (Pa), $\dot{ɣ}$ is the shear rate (s^−1^), *K* and n are parameters of the Herschel–Bulkley model, and ${\dot{ɣ}}_{max}$ (s^−1^) is the maximum shear rate applied.

#### 2.6.3. Compression Test

To carry out the compressive strength tests for each composition and evaluate aging affects three cylindrical specimens of 24 mm in diameter and 28 mm in height were molded. The specimens were demolded after 24 h and immersed in water until the test age. The top and bottom faces were sawed using a low-speed precision saw cutter (IsoMet, Buehler, Grafenberg, Germany) for surface regularization.

The compressive strength of the pastes was determined at 1, 3, and 28 days in cylindrical specimens. For each composition and age, three specimens were tested. The specimens were tested in a universal Instron with a loading rate of 0.50 MPa/s according to ASTM C-1231 [25]. The water absorption and volume of permeable voids of cement pastes at 28 days of hydration were evaluated according to ASTM C642 [26]. Two specimens were tested, and the average was adopted. The binder intensity index (BI) proposed by Damineli et al. [27] is used to assess the binder efficiency. It corresponds to the amount of binder per m^3^ of material produced for a unit of compressive strength (i.e., kg of binder/m^3^·MPa) at a given age. However, the OSW powder used in this study was not considered a binder. Thus, the cement intensity index (CII) corresponds to the consumption of cement per m^3^ of material to achieve 1 MPa of compressive strength [28]. For statistical analyses, the analysis of variance (ANOVA) was conducted using the OriginPro 8.5 (OriginLab, New York, NY, USA) software using two data sets: the OSW incorporation level and the testing age. In addition, a piece from the broken surface of a 28-days specimen of the reference and 10 wt.% OSW pastes was collected, gold-coated, and analyzed by SEM.

### 2.7. CO_2_ Emission Analysis

The CO_2-eq_ relative to 1 m^3^ of paste was estimated for each composition investigated. A corresponding index is obtained by multiplying the amount of each constituent by its respective CO_2-eq_ emission per kg of material. The adopted emissions value was 0.892 kg CO_2-eq_/kg of OPC [29]. For OSW, an equivalent emission of 0.003 kg CO_2-eq_/kg was adopted, which corresponds to the process of grinding. Considering the average value of grinding OSW in ball mill [30], 0.023 kWh/kg of the material was necessary. According to the CO_2-eq_/kWh emissions proposed by IPCC Working Group III—Mitigation of Climate Change [31] for the energy sources of the Brazilian electric power supply matrix (hydropower, biomass, solar and wind power, coal, petroleum products, natural gas and nuclear), 1 kWh is associated with 0.135 kg CO_2-eq_ [32]. The CO_2_ emissions related to the water are very low and are not considered [33,34]. It is worth mentioning that the CO_2_-eq/m^3^ of paste tends to be higher than those of concrete reported in the literature since the CO_2_ emissions associated with cement are much higher than those associated with aggregates. Thus, the CO_2-eq_ associated with 1 m^3^ of concrete was estimated, considering a usual paste volume of 30% of the total volume of concrete [35]. The adopted values of emissions were 0.014 kg CO_2-eq_/kg of fine aggregate and 0.046 kg CO_2-eq_/kg coarse aggregate [36]. The CO_2_ intensity index of the pastes was also calculated, which corresponds to the ratio between the CO_2-eq_ emission of the material and its compressive strength at a given age [27].

## 3. Results

### 3.1. Characterization of Materials

**Figure 2:**
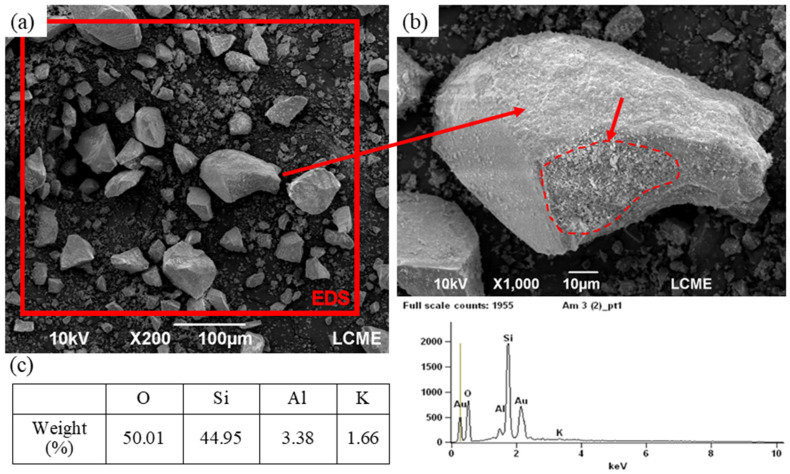
SEM images of the OSW filler at (**a**) ×200 and (**b**) ×1000 magnification, and (**c**) EDS result from OSW filler.

Table 1 shows the chemical composition and the main physical properties of the powder materials. Figure 2 shows the OSW. In Figure 2a, particles of varying sizes and shapes are noted. The large size particles were identified as quartz, hard particles with low grindability. In Figure 2b, clay mineral sheets covering the larger particles are observed. This corroborates EDS results shown in Figure 2c and the chemical composition in Table 2.

**Figure 3:**
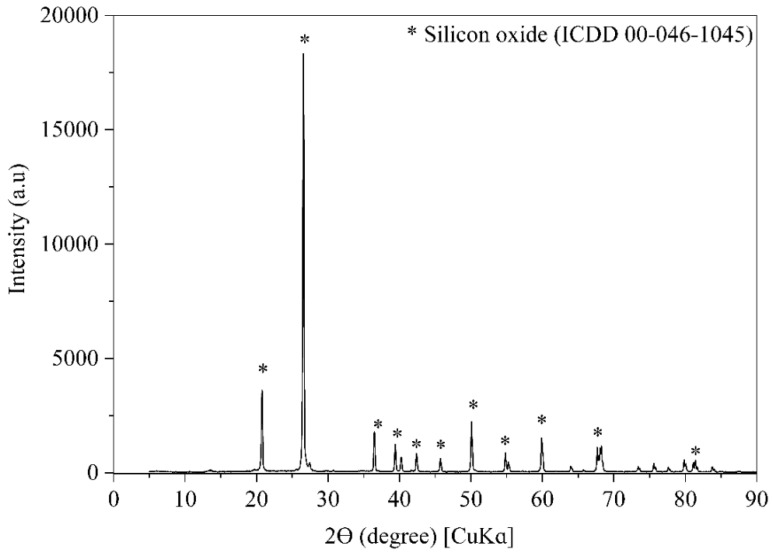
XRD pattern of OSW.

**Figure 4:**
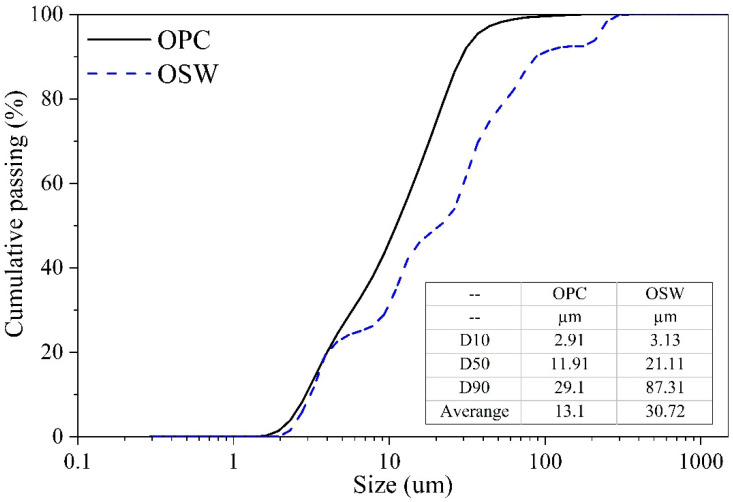
Particle size distribution of OPC and OSW.

Figure 3 presents the XRD patterns of the investigated OSW. The predominant crystalline phase identified by the ICDD is silicon oxide. Furthermore, the XRD pattern shows that the OSW is a crystalline material, indicating low reactivity, justifying its application as a filler as further discussed. The particle size distribution of the OPC and OSW, recorded using a Microtrac S3500 laser granulometer (York, PA 17406, USA) in air condition, is shown in Figure 4.

### 3.2. Isothermal Calorimetry

**Figure 5:**
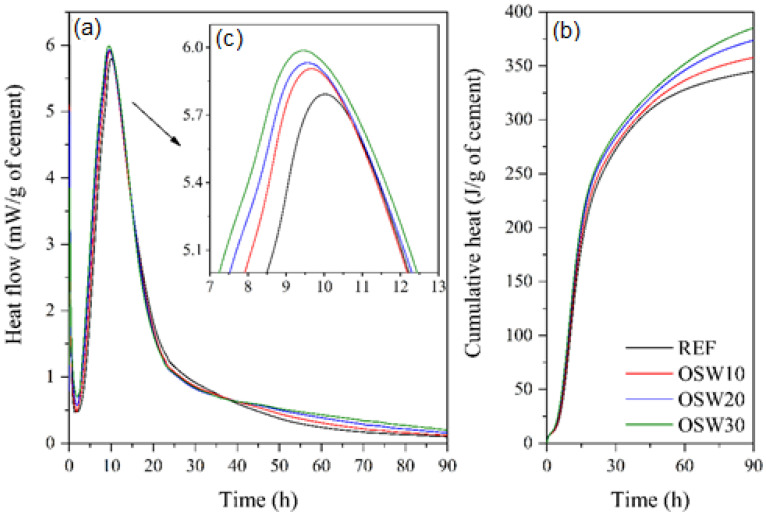
Hydration kinetics. (**a**) rate of heat evolution, (**b**) cumulative heat curves of the pastes, and (**c**) amplification of the main heat peak.

Figure 5 shows the heat evolution curves of all pastes investigated. The values were normalized for the cement mass of the samples. It was observed that the main heat flow peak slightly increased with the increase in OSW content up to 30 wt.%. The main peak of heat (dominated by the growth of hydrates C–S–H and portlandite [37]) increased from 5.79 mW/g (REF) to 5.91–5.99 mW/g (OSW10–OSW30). Furthermore, as the replacement level increased, the 90-h cumulative heat increased. The incorporation of OSW resulted in increases in cumulative heat from 347 J/g (REF) to 389 J/g (OSW30). This increase indicates a slight improvement in cement hydration due to the presence of the sandstone. Besides that, the increase in OSW content progressively anticipated the main peak heat release from 10:02 h (REF) to 9:28 h (OSW30). On the first day, the enhancement of cement hydration is due to the filler effect, which can be attributed to the dilution effect [37]. The reduction in cement volume due to OPC replacement by OSW results in a greater amount of water and space available for its hydration, which leads to an increase in the hydration rate [38,39].

### 3.3. Rheology

**Figure 6:**
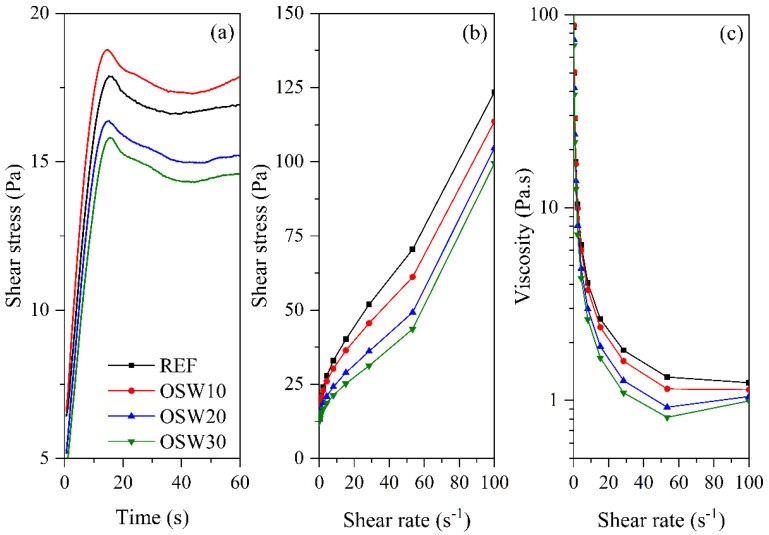
Curves of rheological tests. (**a**) static yield stress determination (**b**) flow curves, and (**c**) viscosity vs. shear rate curves of cement pastes.

Figure 6a shows the shear stress vs. time curves for static yield determination. The shear stress linearly increases until it reaches up a peak, and, subsequently, the peak is followed by a slight decrease in shear stress. This peak defines the static yield stress and indicates the destructuring of the material under slow shear in the liquid regime [40,41]. Figure 6b exhibits the decreasing shear stress vs. shear rate curves, also designated as flow curves. Figure 6c shows the viscosity vs. shear rate curves. Two rheological profiles can be identified in the cement pastes evaluated. REF and OSW10 cement pastes showed: (i) a shear-thinning response at low shear rates, which corresponds to a viscosity decrease with the shear rate increase; and (ii) an approximately constant viscosity at high shear rates. In contrast, OSW20 e OSW30 showed (i) a shear-thinning response at a low shear rate and (ii) a shear-thickening response at high shear rates, which corresponds to an increase in viscosity with the shear rate increase [42]. According to Jiao et al. [43], most cement-based materials have a shear-thinning response at lower shear rates and a shear-thickening response at higher shear rates. In this context, the shear-thickening response can be attributed to either an order–disorder transitions theory or a cluster theory [42]. According to Maybury and Ho [44], the polycarboxylate superplasticizer (SP) can form clusters at high shear rates, causing the shear-thickening behavior of cement pastes. This hypothesis is reinforced by the SP content presented in Table 2. OSW20 and OSW30 cement pastes required a higher SP amount to keep the mini-slump fixed, which may help to explain the change in curve profiles at high shear rates when compared to the REF and OSW10 pastes. Note that this shear-thickening response of cement-based materials can cause some problems for some specific applications. This response can reduce the pumping distance, increase the mixing heterogeneity, and hamper the material handling [44].

**Figure 7:**
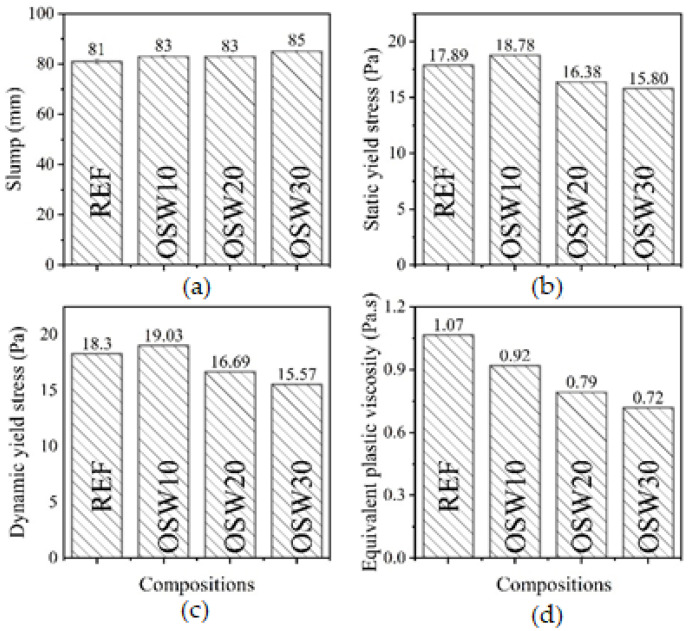
Rheological properties of the pastes. (**a**) Slump; (**b**) static yield stress; (**c**) dynamic yield stress; (**d**) equivalent plastic viscosity.

Figure 7 shows the rheological properties of the cement pastes evaluated. OSW10 exhibited similar static and dynamic yield stresses compared to REF. In turn, OSW20 and OSW30 showed reductions in the yield stress compared to the plain cement pastes, with decreases up to 15.0%. One aspect that affects OSW pastes’ rheological behavior is waste specific gravity. Since OSW has lower specific gravity than Portland cement (see Table 2), it results in a higher solid volume fraction. Nevertheless, the rheological results observed in this study are consistent with the granulometry results previously presented. Considering that the sandstone has a higher particle size than OPC, this yield stress behavior is expected, especially for higher incorporation of OSW contents, e.g., OSW20 e OSW30. This is because the average particle diameter of OSW is higher than OPC, which reduces the adhesive and frictional forces among particles. Thus, it seems that the OSW particle size has a more pronounced effect on rheological parameters than the greater solid fraction volume of the waste associated with lower specific gravity. Furthermore, it should be considered that the OSW cement pastes were composed of higher contents of SP admixture, which also helps to explain the trend observed in the rheological parameters of OSW compositions.

In this context, the yield stress corresponds to the minimum shear stress that makes the material flow or deforms [45]. According to Jiang et al. [45], significant increases in yield stress may hinder matrix compaction. Thus, this can increase the matrix porosity [46]. Therefore, OSW can improve the fresh properties of cement pastes. A similar trend was observed for the equivalent viscosity. Overall, the studies that evaluated the rheological behavior of cement-based materials with ornamental waste are scarce, specifically regarding sandstone waste, limiting the comparison of the results obtained in this research with existing works in the literature. One of the few studies is that of Corinaldesi et al. [47], indicating that replacement contents of OPC by marble waste increased the plastic viscosity of cement pastes with w/c of 0.4 and 0.5 after 15 min of mixing. Nevertheless, it should be noted that the rheological behavior of cement-based materials with ornamental stone waste is highly dependent on the particle size of the waste used and the replacement content.

**Figure 8:**
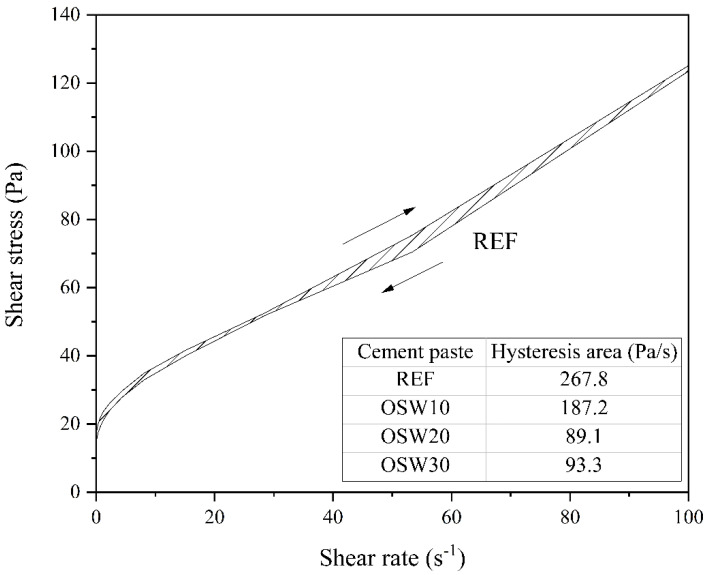
Example of hysteresis area determination of cement pastes evaluated.

The thixotropy is an intrinsic characteristic of cement-based materials related to the colloidal flocculation and cement hydrates nucleation [48]. The thixotropy is associated with the hysteresis area, which can be calculated from the area difference between the increasing and decreasing flow curves, as exemplified for the plain cement pastes (REF) in Figure 8. Note that the hysteresis area values of the other evaluated pastes are presented in the table inserted in Figure 8. Overall, the OSW incorporation reduced the hysteresis area of cement pastes, regardless of the replacement content. The reductions were 30.0% (OSW10), 66.7% (OSW20), and 65.2% (OSW30), compared to the plain OPC pastes (REF). Thus, the OSW with larger particle size reduced the inter-particle contacts and, consequently, reduced the hysteresis area [43]. In this context, according to Jiao et al. [43], a higher thixotropy degree can cause problems on the resuming pumping and the interface behavior between multi-layers. Therefore, considering the results obtained, the OSW incorporation can be interesting for the previously mentioned specific applications.

### 3.4. Compressive Strength Results

**Figure 9:**
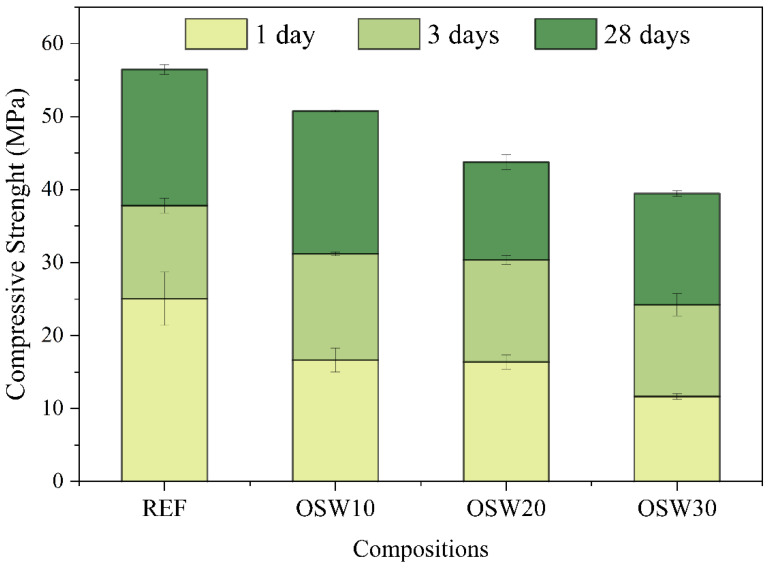
Compressive strength of cement pastes at 1, 3, and 28 days.

Figure 9 shows the compressive strength of the pastes at 1, 3, and 28 days of cure. Table 3 presents the variance analysis (ANOVA) of the compressive strength results. At 1 day, the REF paste presented the highest compressive strength (25 MPa), and with the increase in the OSW content, the compressive strength decreased. This was expected for this age since only a slight improvement in cement hydration due to the presence of OSW was verified in the isothermal calorimetry results. At 3 days, OPC replacement by OSW decreases the compressive strength by 36% from REF to OSW30. At 28 days, the trend was the same. The incorporation of OSW decreases the compressive strength from 56 MPa (REF) to 39 MPa (OSW30).

**Figure 10:**
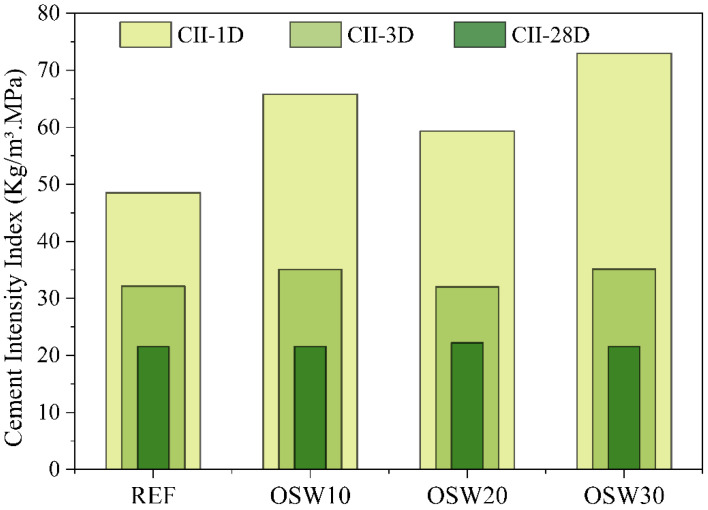
Cement intensity index of the cement pastes at 1, 3, and 28 days of age.

The cement intensity index (CII) of the pastes is shown in Figure 10. At 1 day, of age the incorporation of OSW resulted in an increase in the CII from 48.5 (REF) to 65.8 (OSW10), 59.3 (OSW20), and 73.0 (OSW30). This increase is due to the higher compressive strength of REF at this age, comparing 25 MPa of REF vs. 11–16 MPa for the others. However, at 3 and 28 days of age, despite the reduction in the compressive strength, the compositions with OSW showed CII values equal to the REF, with an average of 33.6 kg/m^3^·MPa and 21.7 kg/m^3^·MPa, respectively.

**Figure 11:**
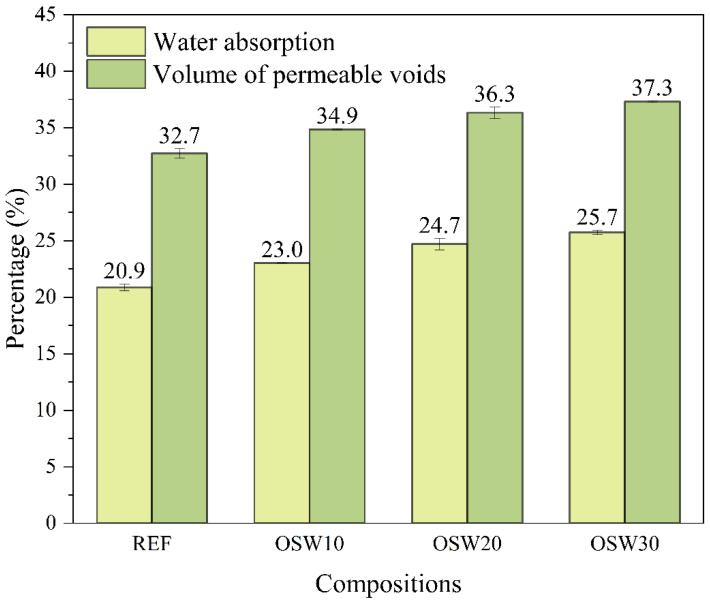
Water absorption and volume of permeable voids of cement pastes at 28 days.

Figure 11 shows the water absorption after immersion and boiling and the volume of permeable voids of cement pastes at 28 days of hydration. Overall, the increase in OSW incorporation content gradually increased both water absorption and volume of permeable voids. Compared to the plain cement paste (REF), increases of 10.0% (OSW10), 18.2% (OSW20), and 23.0% (OSW30) were observed in the water absorption. For the volume of permeable voids, these increases were 6.7% (OSW10), 11.0% (OSW20), and 14.1% (OSW30), respectively. These results agree with the 28-days compressive strength results presented in Figure 9. The water absorption rate indicates cement materials’ quality and potential durability. High water absorption values can indicate the facility of aggressive ions penetration [49]. Therefore, a replacement of 10% of OPC by OSW can be carried out without impairing the compressive strength and porosity of the cement matrix. Moreover, this contributes to the sustainability of the building construction sector, by reducing the OPC consumption. In this context, according to Galetakis and Soultana [50], most studies concerning the use of quarry and ornamental stone waste evaluated the mechanical performance of the specimens. In turn, few studies have evaluated the durability of these materials. Thus, the authors emphasize that durability should be further investigated.

Kumar et al. [3] assessed the water absorption in concretes replacing 0%, 20%, 40%, 60%, 80%, and 100% of natural coarse aggregate by quartz sandstone. The authors observed a gradual increase in water absorption with the increase in quartz sandstone replacement content, which is related to the higher absorption of sandstone aggregates compared to the natural ones. Similar results were reported by Arif et al. [1], which evaluated concretes with sandstone slurry as partial replacement of the total aggregates, i.e., coarse and fine aggregates. Concerning the influence of other ornamental stone wastes as an OPC partial replacement on the water absorption of cement-based materials, few studies have evaluated this property [51,52,53]. Rashwan et al. [51] observed an increase in water absorption and porosity with increasing the replacement amount of OPC by marble and granite in concretes. The authors found that at 28 days, the water absorption increased were between 2.7% and 47.4% compared to the control mix. Almada et al. [52] evaluated 20% cement replacement by ornamental stone waste with varied compositions in cementitious composites. The authors reported that although the water absorption by immersion at 28 days increased between 6.7% and 17.1% with the waste incorporation, the values obtained are within the limit for good quality concrete. Sardinha et al. [53] did not observe expressive differences in the water absorption by immersion of concretes with superplasticizer and the incorporation of marble waste contents of 5%, 10%, and 15%, compared to the control mix. According to the authors, this trend can be attributed to the lower filler effect associated with the particle size distribution of the marble waste.

**Figure 12:**
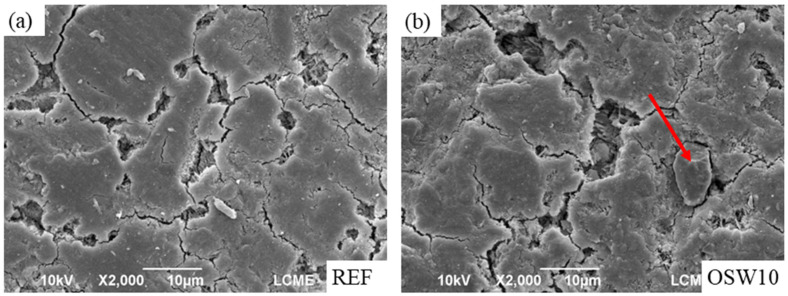
SEM images of (**a**) the REF paste and (**b**) the OSW10 paste.

Figure 12 shows the SEM images of the REF and OSW10 pastes at 28 days of hydration. Figure 12a presents the plain paste without OSW. In Figure 12b, an OSW particle (indicated by the arrow) evolved with the cement matrix is noted. Although there are no substantial changes in the OPC matrix from a visual analysis of SEM images, large voids are observed in the paste with OSW. These observations corroborate the slightly lower compressive strength and the higher water absorption results in pastes containing OSW.

### 3.5. CO_2_ Emission Analysis

Table 4 shows the proportions to produce 1 m^3^ of each paste investigated, as well as the CO_2-eq_ emission related to its constituents and the total CO_2-eq_ emission per m^3^ of paste. The replacement of OPC by OSW progressively decreased the CO_2_ emission of the pastes, from 1084.4 to 743.4 CO_2-eq_/m^3^, corresponding to a reduction of up to 31%. Furthermore, OSW30 paste allows incorporating 356.6 kg of residue per m^3^.

The emissions of concretes produced with the investigated pastes would decrease from 325.3 CO_2-eq_/m^3^ of concrete (REF) to 290.7–223.0 CO_2-eq_/m^3^ of concrete (OSW10–OSW30). These results are below the values reported by other studies for concrete. Alsubari et al. [54] reported 250–450 CO_2-eq_/m^3^ of concrete with partial replacement of Portland cement by palm oil fuel ash. Gursel et al. [28] found 284–544 CO_2-eq_/m^3^ for concrete with OPC mixed with rice husk ash, fly ash, and limestone.

**Figure 13:**
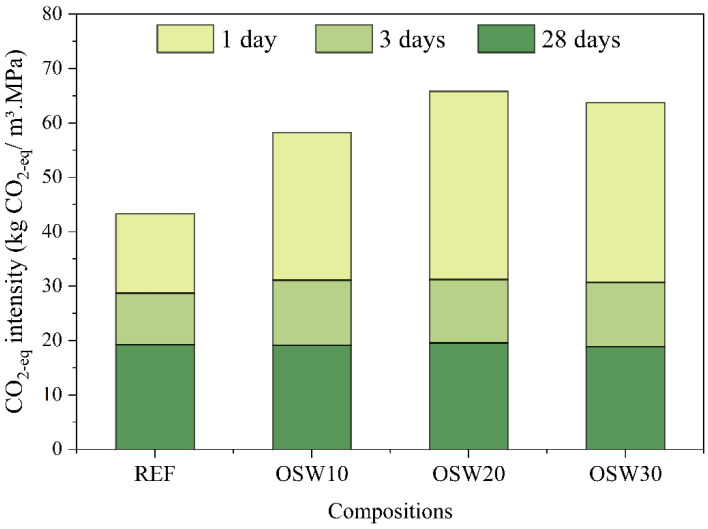
CO_2-eq_ intensity (kg CO_2-eq_/MPa·m^3^ of paste) at each age.

Figure 13 shows the CO_2-eq_ intensity of all compositions of pastes, comparing the ratio between the CO_2-eq_ emission of the material and its compressive strength at 1, 3, and 28 aging days. At 1 day, all OSW mixtures presented CO_2-eq_ intensity higher than REF. This is due to the negative effects of OSW on the compressive strength at this age, despite the contribution of OSW towards CO_2_ emissions. At 3 days, the replacement of OPC by OSW increased the CO_2-eq_ intensity of the pastes, from 28.7 to 31.1 (OSW10), 31.2 (OSW20), and 30.7 (OSW30), corresponding to an increase of 8.0, 9.0 and 7.0%, respectively. At 28 days of age, despite the lower compressive strength of the pastes with OSW, the CO_2-eq_ intensity of the pastes with OSW (18.8–19.5 kg CO_2-eq_/MPa·m^3^ of paste) was equivalent to the CO_2-eq_ intensity of the REF (19.2 kg CO_2-eq_/MPa·m^3^ of paste).

## 4. Discussion

This work investigated the effect of an ornamental stone waste (OSW) incorporated in cement pastes as a partial replacement for ordinary Portland cement (10, 20, and 30 wt.%). As noted in the introduction to this article, the content of OWS affects the performance of cementitious materials. Furthermore, the physical-chemical properties of OSW depend on its origin, influencing the properties of materials produced with the OSW. The present findings revealed differences in cement pastes performance according to the replacement level of OPC by an OSW from southern Brazil.

The knowledge of rheological results has practical applications. Increases in yield stress can mean difficulty on matrix compaction [45]. Our results (Figure 7) showed that pastes with 10% replacement of OPC by OSW present similar yield stress compared to REF. In turn, pastes with 20% and 30% of replacement reduced the yield stress compared to the plain cement paste, improving the pastes fresh properties. These results are consistent with the higher particle size of OSW than OPC (Figure 4).

In pastes with OPC replacement of 10, 20 and 30% wt.%, 1, 3, and 28 d mechanical strength values were lower than REF (Figure 9), a finding consistent with a slight improvement in cement hydration due to the presence of OSW (Figure 5) and the porosity results obtained at 28 days (Figure 11). Similar strength results are reported by other works, which observed a decrease in compressive strength of self-compacting concrete when increasing the replacement content of Portland Pozzolana cement by sandstone, even in long-term ages [2,5,13]. Thus, these results suggest that, due to the inert character of the OSW waste evaluated, higher incorporation (>10.0 wt.%) into OPC by OSW causes significant reductions in the mechanical performance of cement-based materials. Thus, the results indicate that the OSW waste has the potential to be used as a cement filler.

Due to pastes lower compressive strength, the incorporation of OSW increased the cement intensity index compared to REF at this age (Figure 10). However, at 3 and 28 days, the pastes with OSW showed this index equal to the plain paste. This find contributes to the sustainability of cementitious materials by reducing the OPC consumption per m^3^ of material to achieve the same strength level. A similar trend was observed in the CO_2_ intensity index (Figure 13). OSW pastes showed higher values at early ages and similar to REF at 28 days. That is, despite the lower strength, at 28 days, the production of pastes with OSW causes similar CO_2_ emissions for the same level of strength.

Overall, OSW may replace OPC up to 10% to produce sustainable cementitious materials, reaching comparable fresh and hardened properties to plain cement paste. Therefore, due to the OSW inert character, it has the potential to be used as filler in cement-based materials, which also represents a large-scale application for the waste generated in large amounts by the ornamental sone industrial sector. This study was limited to investigating OPC pastes with cement replacement with sandstone from southern Brazil. Future studies can contribute to this topic by investigating sandstone’s effect from other regions since the stone properties may vary, affecting the OPC pastes performance.

## 5. Conclusions

Based on the results presented in this paper, the following conclusions were drawn:

- The results of isothermal calorimetry showed that with the increase in OSW content, the 90-h cumulative heat increased, and the main peak heat release occurred before. This indicates that OSW slightly improved the OPC hydration.
- Rheology results indicated that OSW can improve the fresh properties of cement pastes. This behavior can be attributed to the slightly higher average particle size of OSW compared to OPC particles and to the higher superplasticizer (SP) content of cement pastes with OSW incorporation.
- Pastes with 10% of OPC replacement by OSW can be produced without significantly impacting the compressive strength and porosity.
- The CO_2_ emission per m^3^ of pastes progressively decreases with the increase in the cement replacement content, corresponding to a reduction of up to 31%. The CO_2_ intensity (i.e., kg CO_2-eq_/m^3^·MPa) of the pastes with OSW at 28 days (18.8–19.5 kg CO_2-eq_/MPa·m^3^ of paste) were equivalent to plain cement paste (19.2 kg CO_2-eq_/MPa·m^3^ of paste).

## Figures and Tables

**Table 1:** Consumption of materials per m^3^ of paste.

Mix	Kg/m^3^			
	OPC	OSW	Water	SP
REF	1215.69	0.00	607.84	0.30
OSW10	1094.12	121.57	607.84	0.61
OSW20	972.55	243.14	607.84	0.94
OSW30	850.98	364.71	607.84	1.22

**Table 2:** Chemical composition and physical properties of the powder materials.

Properties	OPC	OSW
Chemical composition (%)	-	-
Al_2_O_3_	4.40	-
SiO_2_	18.62	95.80
Fe_2_O_3_	3.00	0.49
CaO	61.24	0.17
MgO	3.80	-
SO_3_	3.08	-
K_2_O	-	1.06
BaO	-	0.15
Loss on ignition	3.41	1.09
Insoluble residue	0.94	-
Physical properties	-	-
Specific Gravity (g/cm^3^)	3.10	2.60
Fineness (m^2^/g)—BET	2.22	-
d50 (µm)	11.91	21.11

**Table 3:** Two-way ANOVA of compressive strength results.

	Degrees of Freedom (DF)	Sum of Squares (SS)	Mean Square (MS)	F Value	*p* Value	Sig ^a^
OSW content	3	4630.8230	2315.4115	770.4844	0	S
Age	2	873.6204	291.2068	96.9030	3.3984 × 10^13^	S
Error	23	69.1182	3.0051			
Total	28	5566.5267				

^a^ S—Significant.

**Table 4:** Composition and CO_2_-eq emission of 1 m^3^ of paste.

Mixtures	Cement (kg/m^3^ of Paste)	OSW (kg/m^3^ of Paste)	CO_2-eq_/m^3^ of Paste	CO_2-eq_/m^3^ of Concrete *
REF	1215.7	0.0	1084.4	325.3
OSW10	1085.9	120.7	969.0	290.7
OSW20	958.1	239.5	855.4	256.6
OSW30	832.2	356.6	743.4	223.0

* The concrete composition was estimated, considering the paste as 30% of the volume, as per [35].

## Data Availability

Data sharing not applicable.
